# multiSMD – A Python Toolset for Multidirectional Steered Molecular Dynamics

**DOI:** 10.1021/acs.jcim.5c01742

**Published:** 2025-10-02

**Authors:** Katarzyna Walczewska-Szewc, Beata Niklas, Kamil Szewc, Wiesław Nowak

**Affiliations:** † Institute of Physics, Faculty of Physics, Astronomy and Informatics, Nicolaus Copernicus University in Toruń, ul. Grudziądzka 5, 87-100 Toruń, Poland; ‡ Department of Animal Physiology and Neurobiology, Faculty of Biological and Veterinary Sciences, 49577 Nicolaus Copernicus University, Lwowska 1, 87-100 Toruń, Poland; § ESS Engineering Software Steyr GmbH, Berggasse 35, 4400 Steyr, Austria

## Abstract

Molecular forces govern all biological processes from cellular mechanics to molecular recognition events. Understanding the direction-dependence of these forces is particularly critical for elucidating fundamental interactions, such as protein–protein binding, ligand dissociation, and signal mechanotransduction. While steered molecular dynamics (SMD) simulations enable the study of force-induced transitions, conventional single-direction approaches may overlook anisotropic mechanical responses inherent to biomolecular systems. Therefore, probing the mechanical stability of molecular systems with respect to a director of an external force may provide critical information. Here, we present multiSMD, a Python-based tool that automates the setup and analysis of multidirectional SMD simulations in NAMD and GROMACS. By systematically probing forces along multiple spatial vectors, multiSMD captures direction-dependent phenomena, such as changing energy barriers or structural resilience, that remain hidden in standard SMD. We demonstrate the utility of our approach through three distinct applications: (i) anisotropic unbinding in a protein–protein complex, (ii) search for ligand dissociation pathways dependent on the pulling direction, and (iii) force-induced remodeling of intrinsically disordered regions in proteins. multiSMD streamlines the exploration of nanomechanical anisotropy in biomolecules, offering a computational framework to guide experiments (e.g., atomic force microscopy – AFM or optical tweezers) and uncover mechanistic properties inaccessible to single-axis methods.

## Graphical abstract

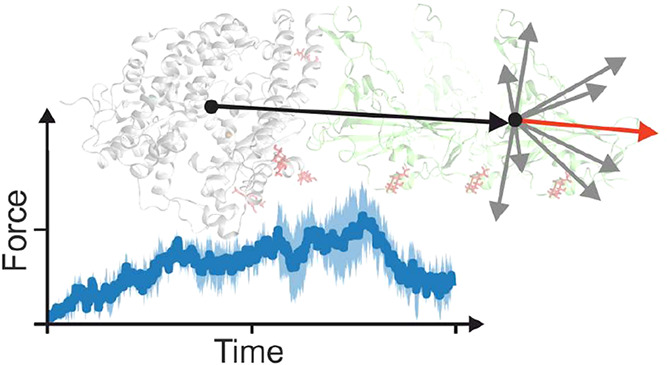

## 1 Introduction

Forces and their temporal evolution are fundamental to the functionality and regulation of living systems, even at the molecular scale. For example, proteins experience and exert forces that drive essential biological processes, including chemotaxis, replication, transcription, translation, protein folding, signaling, ligand binding, cellular transport, and enzymatic catalysis. These forces are attributed to molecular interactions (electrostatic, van der Waals, hydrogen bonding, and hydrophobic effects), and their modulation over time enables structural responsiveness to environmental stimuli and biochemical signals. In fact, the majority of those processes are inherently anisotropic, meaning that their mechanical and dynamic properties vary depending on spatial direction of dominant interactions, due to the complex, nonspherical structures of biomolecules. Understanding the time-dependent and direction-dependent nature of major forces is critical for elucidating mechanisms such as mechanotransduction, allosteric regulation, and binding/unbinding events. Calculations of multidimensional free energy landscapes that govern those forces are notoriously difficult and expensive for large biomolecular systems. Monitoring external forces used to induce a hypothetical conformational change is much easier to perform. There is also a direct link between computational experiments and single-molecule force spectroscopy.

The development of advanced methodologies to investigate molecular forces has significantly enhanced our understanding of their role in biological systems. Atomic force microscopy (AFM), particularly in conjunction with force spectroscopy,^1^ has enabled the direct measurement of piconewton-scale forces in single molecules, revealing insights into ligand binding,^2^ antibody–antigen interactions,^2^ protein unfolding,^3^ or enzymatic catalysis.^4^ Similarly, optical and magnetic tweezers apply controlled forces to molecules (often via attached beads) enabling precise manipulation and study of DNA mechanics, motor protein motility, or receptor–ligand dissociation.^5,6^ While single-molecule force spectroscopy experiments performed with AFM or tweezers are immeasurably useful, they are also very challenging. Problems with proper immobilization of biomolecules during sample preparation, single-molecule identification, and discrimination of nonspecific interactions make this technique rather unsuitable for high-throughput investigations.^7^

Computational methods, particularly molecular dynamics (MD) simulations provide a complementary approach, serving as a computational microscope for molecular biology.^8^ In MD simulations, forces acting on each atom of the investigated biomacromolecule are calculated to update their positions in space. Thus, the time evolution of a molecular system at atomic resolution is predicted. In steered molecular dynamics (SMD), time-dependent external forces are applied to the system along the preselected coordinate, usually a straight line, to accelerate transitions between energy minima in the free energy landscape. This nonequilibrium modeling enables studying biophysical processes that require time scales not accessible for classical MD, such as protein (un)­folding,^9^ transport across a membrane,^10^ identification of ligand binding pathways,^11,12^ or elucidation of the dynamics of big protein complexes.^13,14^ SMD also serves as a tool for comparing binding affinities of small-molecule ligands to their target proteins by measuring the force required to pull them out of their binding sites,^15^ thus providing valuable insights into the inhibitory potential of drug candidates. While methods like the Jarzynski equality can be used to estimate free energies from SMD work values,^12^ the present study focuses on a qualitative and comparative analysis of mechanical anisotropy. While traditional SMD simulations focus on a single pulling direction, many biological processes, such as the anisotropic response of proteins to mechanical stress or the directional dependence of ligand unbinding pathways, are best studied through multidirectional force probing.^16^ Such an approach can reveal subtle differences in energy landscapes, hidden transition states, and direction-dependent resistance to deformation, which might otherwise remain undetected in conventional single-axis simulations. Multidirectional SMD simulations can thus complement and guide experiments like AFM and optical/magnetic tweezers by exploring force-dependent processes from multiple angles prior to initiating labor-intensive experimental setups.

To bridge this gap, we developed multiSMD, a tool that automates the preparation of inputs for multidirectional SMD simulations. This Python-based script generates a series of batch files enabling SMD simulations in popular MD engines (NAMD and GROMACS) based on the protein complex structure provided (Figure 1a). To facilitate data postprocessing, multiSMD comes with a dedicated script *analysis*.*py* for analyzing the obtained results. This script allows the user to extract and visualize data relevant to the simulated systems. By enabling systematic exploration of force responses along multiple force vectors, multiSMD facilitates a more comprehensive understanding of anisotropic biological processes such as protein mechanostability, direction-dependent unbinding mechanisms, or the mechanical roles of structural motifs in large complexes.

**Figure 1:**
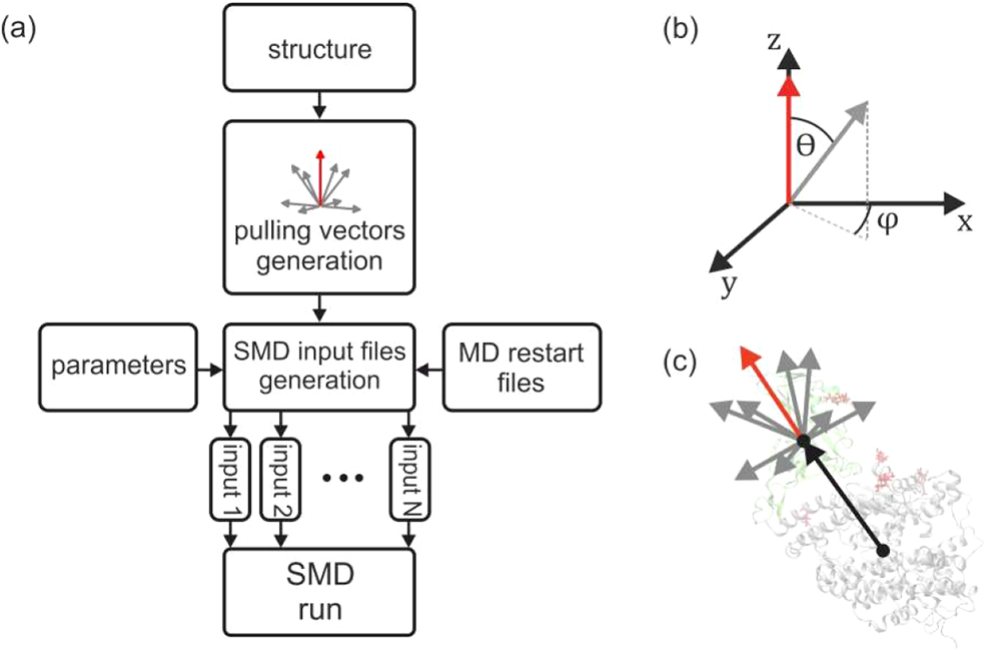
(a) Flowchart illustrating the operation of a multiSMD. (b) Angles describing the successive generated pulling force vectors. (c) Directions of the external force application in parallel SMD simulations of the test system of the S-protein-ACE2 (angiotensin-converting enzyme) model.

## 2 Implementation

The multiSMD programs (multismd_mand.py and *multismd_gromacs.py*) are written in Python 3 and maintained on GitHub (https://github.com/kszewc/multiSMD). The flowchart of each program is shown in Figure 1a. Provided the Cartesian coordinates of protein complex atoms in the PDB format, the program computes the principal axis of ″pulling,” which serves as a vector connecting the centers of mass of the fixed and pulled proteins (see Figure 1c,b). Drawing from this principal axis, multiSMD generates a comprehensive set of vectors, characterized by variations in theta and phi angles within spherical coordinates. Each vector within this set denotes a unique direction for stretching the molecular system during SMD simulations. Notably, users retain the flexibility to adjust the sampling density of this force vector space, with default settings encompassing three exploratory angles (0, 45, and 90 deg) in the theta coordinate and four angles (0, 90, 180, 270 deg) in the phi coordinate. This configuration yields a total of nine distinct pulling directions, effectively covering a selected hemisphere. Upon execution, multiSMD generates an output directory containing the input files and subdirectories corresponding to the ″pull″ directions. Each subdirectory contains an appropriately prepared input files for NAMD/gromacs and a bash script to run the given SMD simulation.

The additional scripts (*analysis_namd.py* for NAMD and *analysis_gromacs.py* for gromacs) allow for extracting essential SMD data from MD output files and obtained trajectories. This includes information about the change in pulling force over time, which allows us to identify the point of the maximum pulling force or maximum mechanical stability along the selected direction. Additionally, the script calculates the dependence of the pulling force on the distance between centers of masses of two predefined atom groups, allowing us to observe the system’s response to the applied force directly. Furthermore, using the MDAnalysis library,^17^ one can analyze the obtained trajectories to monitor formation and breakage of hydrogen bonds between the protein fragments of interest. All of these quantities are plotted using the Matplotlib graphics library. The example output from the multiSMD analysis script, showing the force variation over time, force versus distance, and the time-dependent change in hydrogen bond count due to varying pulling direction is shown as a Supporting Figure S3.

For visualization purposes, the program generates a Tcl script to be used in Visual Molecular Dynamics (VMD) software,^18^ which renders a set of vectors originating from the center of mass and illustrating all specified pulling directions.

## 3 Results and Discussion

We applied our method to the SARS-CoV-2 spike protein-ACE2 complex to examine the anisotropy in mechanical stability, addressing the key question: What forces are required to rupture this protein–protein interaction in multiple directions? Additional case studies investigating (ii) protein–ligand interactions and (iii) systems involving intrinsically disordered regions are presented in the Supporting Information.

We first evaluated the efficacy of our multiSMD program by applying it to the COVID-19 relevant protein system. A key stage of viral infection is the interaction between viral spike proteins and human angiotensin-converting enzyme 2 (ACE2) receptor.^19^ Current drug discovery strategies for COVID-19 focus on weakening these specific protein–protein interactions by blocking the ACE2 receptor or viral Spike protein to prevent infection. We thus proceed to measure the forces required to rupture this complex considering their anisotropy (see Figure 2a for specific directions).

**Figure 2:**
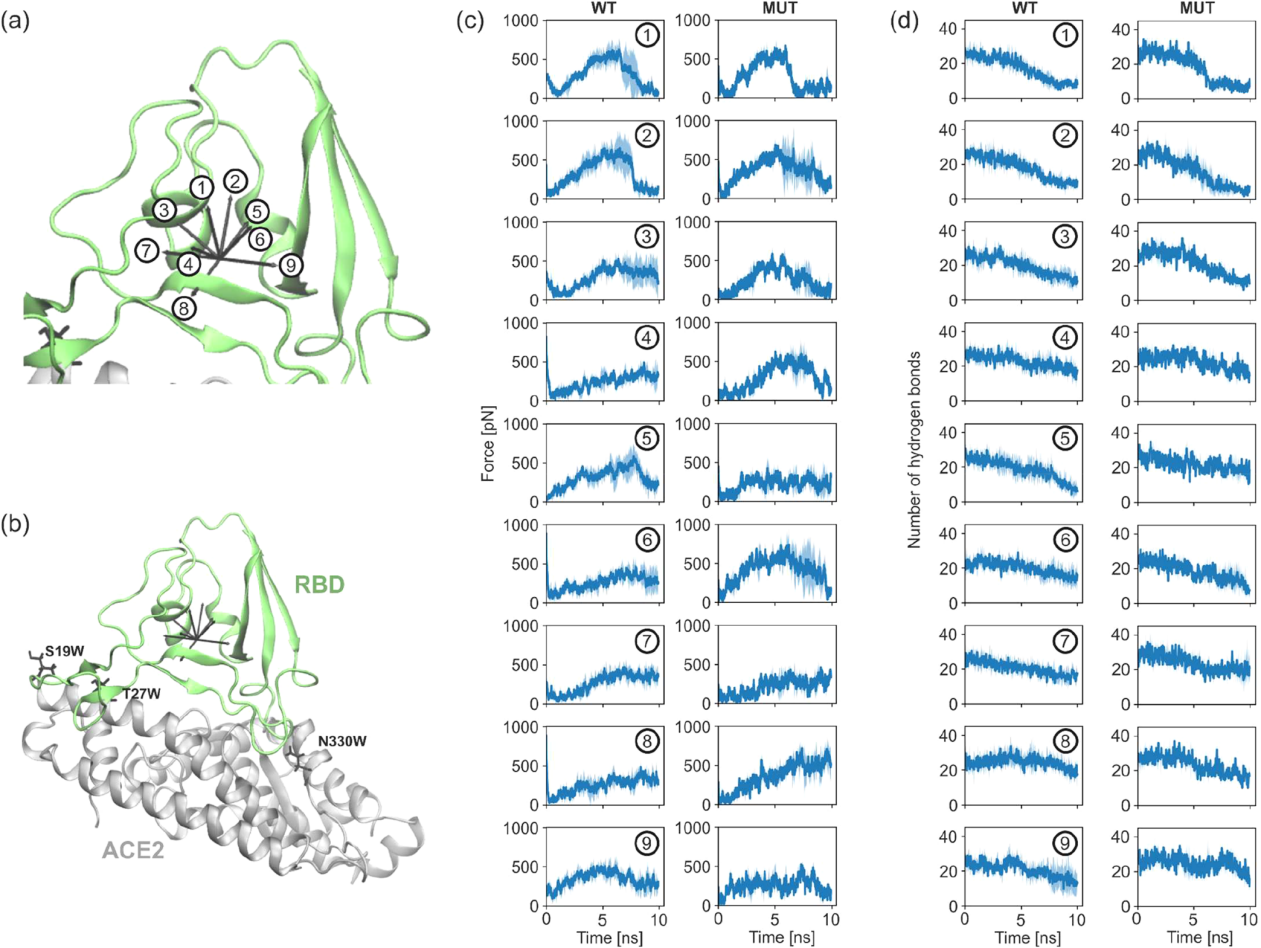
Multidirectional analysis of SARS-CoV-2 S - ACE2 unbinding. (a) Schematic of nine pulling directions applied to the complex. (b) Simulation system of truncated S - ACE2 complex shown in green and gray with mutated residues shown as black sticks. (c) Maximum rupture forces across pulling directions for wild-type (WT) versus mutant (MUT) complexes. (d) Number of hydrogen bonds during S-protein pulling. For panels (c) and (d), the solid lines represent mean values, and the light-blue shaded areas (outline) represent the standard deviation (SD), both calculated from five independent replica simulations for each pulling direction.

We used the structure of the receptor-binding domain (RBD) of the spike protein bound to ACE2 ^20^ (PDB ID 6M0J, see Supporting Figure S4). Using microsecond long MD simulation of the complex,^21^ we were able to extract a structurally stable fragment of the complex interface (see Supporting Information for details). We also performed *in silico* mutagenesis of the ACE2 receptor, focusing on three mutations, S19W, T27W, and N330Y (see Figure 2b), previously shown to enhance SARS-CoV-2 S-RBD binding,^22^ to investigate their impact on SMD pulling forces. The structures of the SARS-CoV-2 S-RBD bound to the ACE2 mutants reveal that the increased binding affinity is mainly due to van der Waals interactions created by the aromatic side chains of W19, W27, and Y330.

After equilibration and 0.25 μs of classical MD simulation using NAMD performed to stabilize the system, we ran our *multismd_name.py* script to generate inputs for SMD simulations in nine directions of pulling (Figure 2a). We ran 5 replicas of 10 ns SMD in each direction for nonmodified complex (APO) and mutated system (MUT). A detailed description of the methods and investigated system is provided in Supporting Information.

We based our analysis on a calculation of (i) the changing number of hydrogen bonds when pulling the RBD in various directions (Figure 2d), and (ii) forces required to rupture the complex (Figure 2c). In a simulation time as short as 10 ns, we observed a significant anisotropy in the complex’s response to external forces. In a system without modifications (WT), pulling in all directions resulted in a reduction in the number of hydrogen bonds. However, when mutations were introduced, the number of hydrogen bonds was constant upon pulling in some directions (see WT 4, 5, and 7 in Figure 2d). Greater forces were also required to rupture the mutated complex, although not in all directions tested. These results suggest stronger interactions between RBD and ACE2 when substitutions of residues with aromatic side chains are present, which may enhance virulence. One should bear in mind that the simulations were performed using only interacting parts of both proteins (not a full system), so the allosteric effects were not possible to capture and only part of protein S – ACE2 interactions were analyzed.

The computational cost of multiSMD scales with the number of directions and replicas. However, its automated workflow enables efficient high-throughput screening of HPC resources. For a detailed cost analysis and optimization strategies, see the Supporting Information.

## 4 Conclusions

Biological systems exhibit fundamental anisotropy in their mechanical responses, yet most computational tools offer the study of molecular interactions along single directions. Our multiSMD approach addresses this limitation by enabling easy systematic, multidirectional force probing of biomolecular systems. Through three representative case studies, we demonstrated how this method provides unique insights into direction-dependent phenomena.

In the SARS-CoV-2 spike-ACE2 system, we observed that stabilizing mutations increased the mechanical resistance preferentially along specific pulling vectors, explaining their enhanced binding affinity. As detailed in the Supporting Information, our comparative studies of Kir6.1/Kir6.2 channels revealed isoform-specific ATP binding mechanics, with up to 1.5-fold differences in unbinding forces depending on pulling direction. Similarly, the supplementary analysis of KNt extraction from the SUR2B pocket further demonstrated how intrinsically disordered regions exhibit path-dependent release mechanisms modulated by small molecules.

The multiSMD toolkit simplifies these analyses through automated workflow generation for major MD packages (NAMD, GROMACS) and integrated analysis tools. While the current implementation focuses on basic force profiling, the modular design permits future expansion to advanced sampling techniques. As an open-source resource, multiSMD aims to make anisotropic force analysis accessible to both specialists and nonexperts. Structures generated during multiSMD simulations may be used as starting points for more advanced studies of free energy profiles along specific classical coordinates.

This approach complements existing experimental techniques by identifying critical pulling directions before laborious AFM or optical tweezer experiments. Looking ahead, we anticipate applications in rational drug design, where understanding direction-dependent binding mechanics could help to optimize therapeutic compounds. The case studies presented here establish multiSMD as a practical tool for probing the directional complexity inherent to biological macromolecules.

## Supplementary Material



## Data Availability

The multiSMD software is made available under the Apache 2.0 license, ensuring accessibility and adherence to noncommercial terms of use. The program, accompanied by comprehensive documentation, is hosted on GitHub at the following repository: https://github.com/kszewc/multiSMD.

## Notes

Two additional case studies: (II) comparative analysis of Kir6.1/Kir6.2 channels and their ATP binding mechanics, (III) KNt release from SUR2B pocket in vascular KATP channels; example multiSMD analysis outputs showing force profiles and structural changes; molecular model of the truncated ACE2-spike protein complex; conformational dynamics analysis (RMSF) of full vs truncated systems; residue-specific contact frequencies between Kir6.1/Kir6.2 and ATP; computational costs and optimization strategies (PDF)
