# Supplementary material for: multiSMD – A Python Toolset for Multidirectional Steered Molecular Dynamics
Source: J Chem Inf Model. 2025 Oct 2;65(20):10803–7. doi: 10.1021/acs.jcim.5c01742 (PMC12570128; doi:10.1021/acs.jcim.5c01742)
Supplement: Supplementary file 1 [file ci5c01742_si_001.pdf]

Supplementary Information: multiSMD – a Python toolset for multidirectional Steered Molecular Dynamics

Katarzyna Walczewska-Szewc [1]*, Beata Niklas [2], Kamil Szewc [3], Wiesław Nowak [1]

1. Institute of Physics, Faculty of Physics, Astronomy and Informatics, Nicolaus Copernicus University in Toruń, ul. Grudziądzka 5, 87-100 Toruń, Poland;
2. Department of Animal Physiology and Neurobiology, Faculty of Biological and Veterinary Sciences, Nicolaus Copernicus University, Lwowska 1, 87-100, Torun, Poland
3. ESS Engineering Software Steyr GmbH, Berggasse 35, 4400, Steyr, Austria
* kszewc@umk.pl

Methods

1. SARS-CoV-2 S protein – ACE2 complex

The COVID-19 relevant protein system involved the receptor-binding domain (RBD) of the spike protein of SARS-CoV-2 virus bound to the cell receptor ACE2 (PDB ID 6M0J[1]). Using trajectory from microsecond-scale MD simulations[2], we isolated the interface region of the protein, consisting of one of the ACE2's claw-like parts and a dynamically distinct fragment of the S protein (Figure S4 and S5). The structure was examined for missing side-chains and refined using Schrodinger software. CHARMM-GUI[3] was used to model glycosyl groups and to create topologies (CHARMM36m force field[4]). The system was embedded into a water box of size 90x90x90 Å and neutralized with KCl. Equilibration followed by 0.25 µs classical MD simulation were run using NAMD 2.14[5]. Output files were read by our script to generate inputs for SMD. Cα atoms of ACE2 were constrained and Cα atoms of Spike S protein were selected to be pulled. 10 ns SMD simulations for nine pulling directions were conducted with five independent replicas performed for each direction. The mutated system was created based on the WT system by substituting three amino acids of ACE2 using the CHARMM-GUI server. The substituted amino acids were S19W, T27W, and N330Y. These amino acids were previously shown to enhance SARS-CoV-2 S-RBD binding[6]. The subsequent steps, including equilibration and SMD simulations, in both systems were performed as described for the unmodified system.

2. ATP unbinding from Kir6.1 and Kir6.2

Molecular dynamics (MD) simulations were performed on closed isoforms of Kir6.1 (PDB: 7MIT) and Kir6.2 (PDB: 6C3P) in the presence of four ATP molecules. Systems were prepared using CHARMM-GUI, with ATP molecules placed in binding sites (optimized via Schrödinger's Preparation Wizard). Each protein was embedded in an asymmetric lipid bilayer (outer leaflet: 100% POPC; inner leaflet: 90% POPC, 10% SAPI24; dimensions: 100 × 100 Å). The CHARMM36m force field was applied. Simulations were conducted in GROMACS 2020, starting with energy minimization, followed by seven equilibration steps, and three independent 250 ns production runs. Temperature and pressure were regulated using the Nosé-Hoover thermostat and Parrinello-Rahman barostat, respectively. Final frames served as starting points for subsequent steered MD (SMD) simulations in the NVT ensemble (Nosé-Hoover thermostat). For each system, three independent replicas of SMD simulations were performed for each pulling direction. Trajectory analysis was performed using custom Python scripts. SMD simulations

3. **KNt Release from SUR2B Pocket in Vascular KATP Channels**

The SUR2B/Kir6.1-Nt (26 residues) complex was modeled based on PDB ID 7MJP using Schrödinger, then embedded in a POPC membrane and solvated in CHARMM-GUI (box dimensions: 135 × 135 × 160 Å). The system was energy-minimized and equilibrated in GROMACS, followed by three independent 200 ns production runs (NPT ensemble, Nosé-Hoover thermostat, Parrinello-Rahman barostat) to relax the system. These trajectories served as starting points for subsequent steered MD (SMD) simulations performed under NVT conditions (Nosé-Hoover thermostat). Three independent replicas of SMD simulations were conducted for each pulling direction.

Case study II: The comparison of ATP unbinding from potassium ion channels fragments Kir6.1 and Kir6.2.

Inward-rectifying potassium (Kir6.x) proteins (Figure S1a) are the pore-forming subunits of ATP-sensitive potassium channels (KATP). These channels regulate the flow of potassium ions, and thus the membrane excitability, based on the energy level of the cell (the ratio of ATP to ADP)[7]. Different combinations of Kir6.1 and Kir6.2 and sulfonylurea receptor (SUR1, SUR2a, and SUR2b) subunits generate various KATP subtypes with distinct tissue distributions and functions[8]. Despite significant sequence and structural similarity, Kir6.1 and Kir6.2 isoforms differ in their sensitivity to ATP[9,10]. The ATP-binding site in both

isoforms is highly conserved, with nearly identical residues involved in ligand interaction, as confirmed by available cryo-EM structures (eg. 6C3P[11] and 7MIT[9]) and our unbiased MD simulations. MD snapshots illustrating ATP bound to Kir6.1 and Kir6.2 are shown in Figure S1c,e. Figure S6 illustrates the frequency of close contacts between individual residues and ATP throughout three unbiased short simulations.

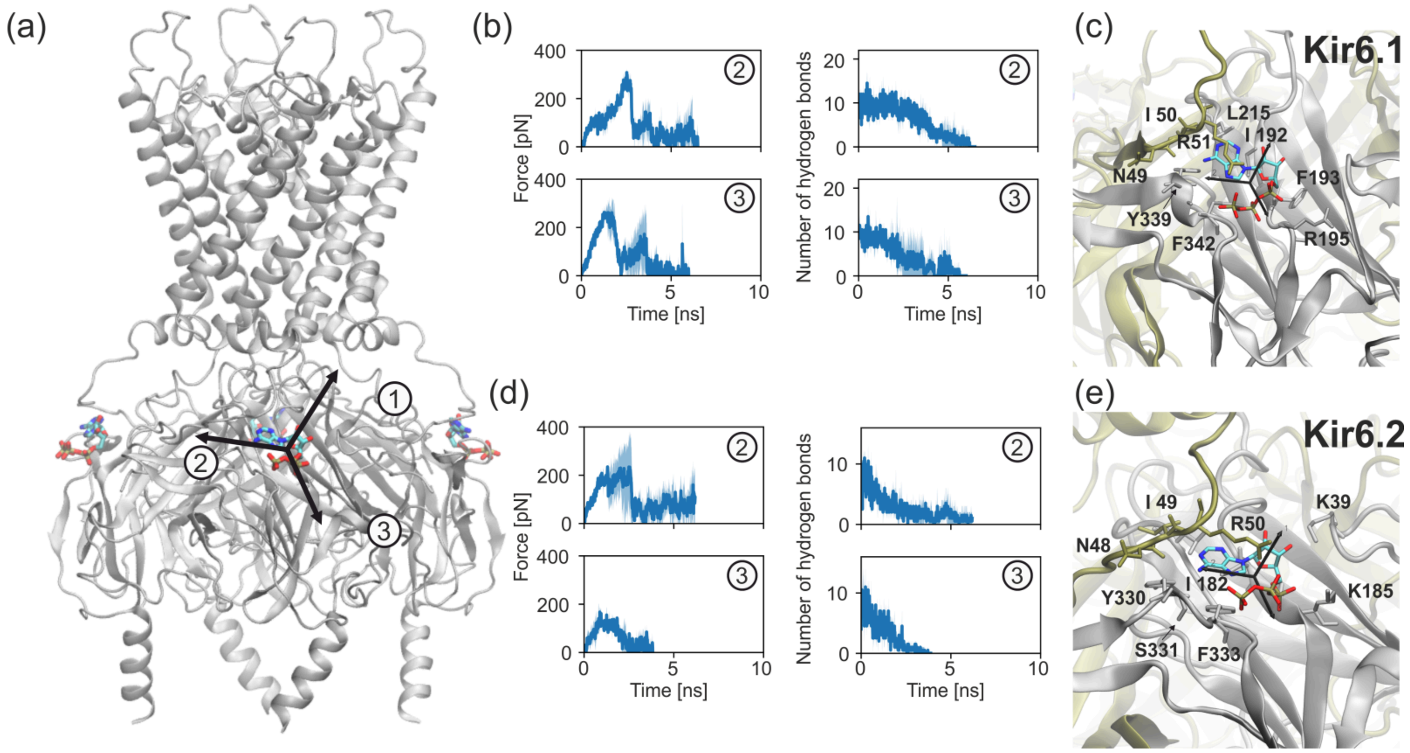

Figure S1. Direction-dependent ATP unbinding from Kir6.1/Kir6.2 channels. (a) Structural overview of Kir6.x tetramer (cartoon) with bound ATP (sticks). (b,d) Force profiles during ATP extraction along direction 2 and direction 3, showing Kir6.1 (b) versus Kir6.2 (d). (c,e) MD snapshots of ATP (cyan) binding sites in Kir6.1 (c) and Kir6.2 (e), highlighting key residues (sticks). For panels (b) and (d), the solid lines represent mean values, and the light-blue shaded areas (outline) represent the standard deviation (SD), both calculated from three independent replica simulations for each pulling direction.

Experimental studies have reported notable differences in ATP-binding between the isoforms despite the high conservation of the ATP-binding site[9,10]. One distinguishing feature is the substitution of R195 in Kir6.1 with K185 in Kir6.2. Both residues are positively charged and critical for ATP binding, yet the substitution may subtly influence ligand interaction. To explore this further, we applied our multiSMD method to evaluate the forces required to extract ATP from the binding pocket at a constant velocity of 0.0005 nm/ps. Starting from equilibrated Kir6.1 and Kir6.2 systems, we pulled ATP along three directions, recording unbinding forces until complete ligand dissociation (three repeats for each pulling direction).

We excluded the first pulling direction from analysis, as the ATP trajectory led to the cell membrane, which has no physiological rationale. Our results show significant differences in forces required for pulling along the third direction, where Kir6.1 requires 1.5 times greater force than Kir6.2. In turn, pulling along the second direction yields similar force profiles for both isoforms (Figure S1b,d). Notably, direction 3 leads ATP toward the region involving the R195/K185 substitution. Analysis suggests that R195 in Kir6.1 forms stronger electrostatic interactions with the triphosphate moiety of ATP than K185 in Kir6.2, which may explain the observed difference. For the third pulling direction, forces required to pull the ATP molecule out of its binding site were higher for Kir6.1 than for Kir6.2, and a greater amount of time was required to break the hydrogen bonds between the Kir6.1 protein and the ligand.

These findings are consistent with the hypothesis that Kir6.1 may exhibit tighter ATP binding compared to Kir6.2, at least in the context of the isolated Kir6 tetramer. However, it is important to note that ATP sensitivity in the full KATP channel complex (including the SUR subunit) is influenced by additional factors, such as interactions with SUR, the presence of Mg-nucleotides, and interactions with PIP2, which

are not captured in our simplified model. For instance, Kir6.1-containing channels require Mg-nucleotides to open and exhibit lower open probability compared to Kir6.2-containing channels, despite potentially stronger ATP binding to Kir6.1[9,10,12]. Thus, while our simulations provide insights into the local differences in ATP-binding mechanics between Kir6.1 and Kir6.2, they do not fully explain the physiological differences in ATP sensitivity observed in the complete KATP channel complex.

These preliminary findings highlight the potential of our multidirectional SMD method for identifying subtle yet functionally significant differences in ligand binding and interaction sites. Further energetic analysis, for example with metadynamics, as well as simulations incorporating the full KATP channel complex, will be needed to confirm these results and provide deeper insights into the interplay between ATP binding, channel gating, and regulation by SUR subunits

Case study III: KNt Release from SUR2B Pocket in Vascular KATP Channels.

The tight interaction between Kir6.x and SUR subunits, which involves both their interfacial contacts and the interactions of disordered regions, is a recently described feature of KATP channels[9,13–16]. In vascular KATP channels (Kir6.1/SUR2B), the N-terminus of Kir6.1 (KNt) strengthens this interaction by docking into a distal pocket of SUR2B. A similar mechanism occurs in pancreatic KATP channels (Kir6.2/SUR1), suggesting a conserved regulatory role. This interaction, through a not yet fully understood mechanism, leads to channel closure. In cryo-EM structures of closed KATP channels, electron densities corresponding to the N-terminus of Kir6 can be observed within this pocket[9,16]. However, the N-terminus is absent from the pocket in open-channel structures where SUR NBD domains are dimerized, suggesting a physiological insertion and removal process[17]. This indicates that KNt should effortlessly exit the pocket when required.
The process of KNt release from the SUR2B pocket is enigmatic. Its directionality is unclear because KNt, an intrinsically disordered region (IDR), lacks a defined position outside the pocket. This makes it an ideal system for testing our multiSMD approach. A simulation system comprises SUR2B with the KNt region of Kir6.1 inserted into the pocket (Figure S2a), representing a fragment of the vascular KATP channel. We started with unbiased MD simulations to equilibrate the system. Two systems were constructed: one without ligands and another with glibenclamide in the pocket. Glibenclamide stabilizes the KNt position and supports the inward-open conformation of SUR2B, corresponding to the KATP channel's closed state[16].
The frequency of close contacts between KNt and SUR residues in the unbiased simulation is presented as bar plots in Figures S2b and S2c (for systems without a ligand and with glibenclamide, respectively). Snapshots illustrating the position of KNt within the pocket and surrounding residues are also shown. We identified two possible pulling directions for KNt for the SMD simulations, denoted as directions 1st and 2nd (Figure S2a). Artificial pulling forces were applied to the proximal part of KNt (residues 20-22), allowing us to evaluate which direction requires less force and, consequently, suggests a more straightforward release pathway.
The profiles of forces required to extract the distal KNt region (residues 1-10) from the pocket in a function of simulation time, are shown in blue plots in Figures S2b and S2c for systems without and with glibenclamide, respectively. The graphs cut off at the point where KNt fully exits the SUR1 pocket. Significant differences between the first and the second pulling directions were observed. For the first direction, a force of approximately 400 pN was initially required to overcome strong interactions between SUR's E1196 and KNt's K24 as well as E1173 and R23. These residues form stable interactions that must be disrupted for the KNt tail to exit the pocket. In this case, the force is applied tangentially to the expected direction of those interactions, which is less effective than applying it perpendicularly. Therefore, pulling along the second direction was initially easier, with resistance increasing as the distal KNt region began leaving the pocket.
Notably, the presence of glibenclamide slightly increased the force required for KNt extraction, particularly in the second direction. While these findings suggest potential pathways and interactions affecting KNt release, more precise methods, such as umbrella sampling or metadynamics, are necessary for a detailed potential of mean force characterization of the process.

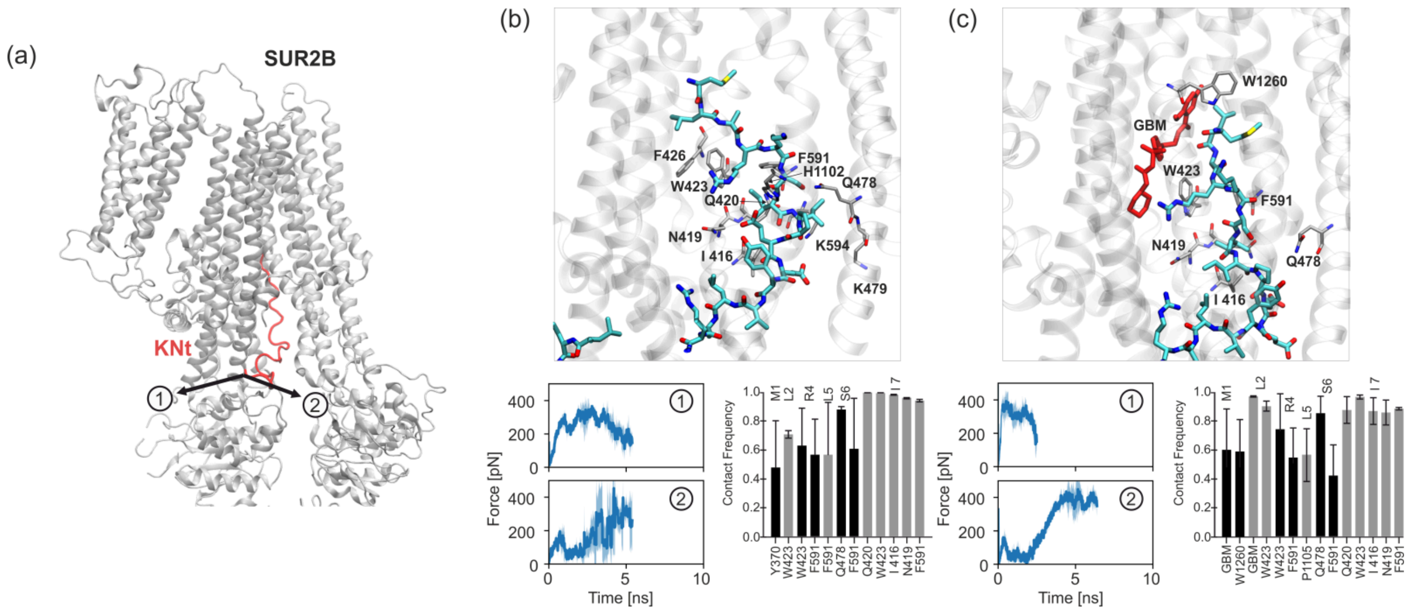

Figure S2: Multi-directional analysis of KNt release from SUR2B. (a) System overview with two tested pulling directions (arrows). (b) System without a ligand: a snapshot of binding pocket (upper, KNt shown as cyan sticks, SUR2B residues are shown as gray sticks), extraction force profiles (lower left), and KNt-SUR2B contact frequencies (lower right). (c) System with glibenclamide: a snapshot of binding pocket (upper, GBM shown in red), extraction force profiles (lower left), and KNt-SUR2B contact frequencies (lower right). For plot insets in panels (b) and (c), the solid lines represent mean values, and the light-blue shaded areas (outline) represent the standard deviation (SD), both calculated from three independent replica simulations for each pulling direction.

Computational Cost and Optimization Strategies

Multi-directional SMD simulations, while powerful for probing mechanical anisotropy, are computationally demanding. The total cost scales linearly with the number of simulated directions, replicas, and system size. The simulations for this study were performed on CPU partitions of two supercomputers: the OKEANOS supercomputer (ICM, Poland) and the LUMI supercomputer (CSC, Finland).

Case Study Resource Requirements:

1. SARS-CoV-2 S-RBD:ACE2 complex (~80,000 atoms, simulated with NAMD 2.13):

Hardware: Computations were performed on the LUMI supercomputer.
Cost per replica: A single 10-ns SMD replica required approximately 38.8 CPU-hours (Wall time: 38.8 hours).
Total cost for the study: The complete study of 9 directions × 5 replicas × 2 variants (WT and MUT) consumed approximately 3,492 CPU-hours.

2. Kir6.1/ATP system (~272,000 atoms) and SUR2B/KNt system (~304,000 atoms), both simulated with GROMACS 2020:

Hardware: Computations were performed on the OKEANOS supercomputer.
Configuration: 5 nodes, 120 CPU cores total (24 cores/node).
Cost per replica: A single SMD replica required approximately 1,837 CPU-hours. Wall time per replica was approximately 7.65 hours.
Total cost for a typical study: A study involving from 2 to 3 directions with 3 replicas each represents a significant but manageable investment on an HPC cluster.

To make such investigations feasible and efficient, we emphasize the following strategies:

1. Screening Approach: The primary strategy is to treat multiSMD as an initial computational screening step. Short simulations (5-20 ns) are used to identify a handful of interesting directions (e.g., those with the highest rupture force or a unique mechanism). Only these selected directions are then subjected to more expensive and precise free energy calculations (e.g., using the Jarzynski equality, metadynamics, or MM-GBSA/PBSA). This approach ultimately saves resources by preventing costly simulations in non-informative directions.

2. Parameter Tuning: The computational cost per simulation can be managed by optimizing SMD parameters:

3. Pulling Velocity: Using a higher velocity (e.g., 0.005-0.01 nm/ns) reduces the simulation time required to observe a rupture event but may overestimate the absolute forces. This is often acceptable for comparative and screening purposes where relative differences between directions are the key outcome.

4. System Size: Careful construction of the simulation system is crucial. Using the minimal physiologically relevant system significantly reduces the number of atoms and cost.

5. Simulation Length: The required length depends on the process. For many unbinding or unfolding events, the core rupture occurs within a few nanoseconds. Pilot simulations can help determine the minimum time needed to observe the event of interest across all directions.

6. High-Throughput Execution: The multiSMD tool automates the generation of hundreds of input scripts. Crucially, simulations for different directions are entirely independent. This makes the workflow ideal for high-throughput execution on large HPC clusters, allowing thousands of core-hours to be used simultaneously and drastically reducing the total wall-clock time required to complete the study.

We acknowledge that cost is a limiting factor. The strategies above, combined with the ever-increasing availability of powerful computing resources, make multi-directional mechanical probing an increasingly accessible tool for uncovering key biophysical insights.

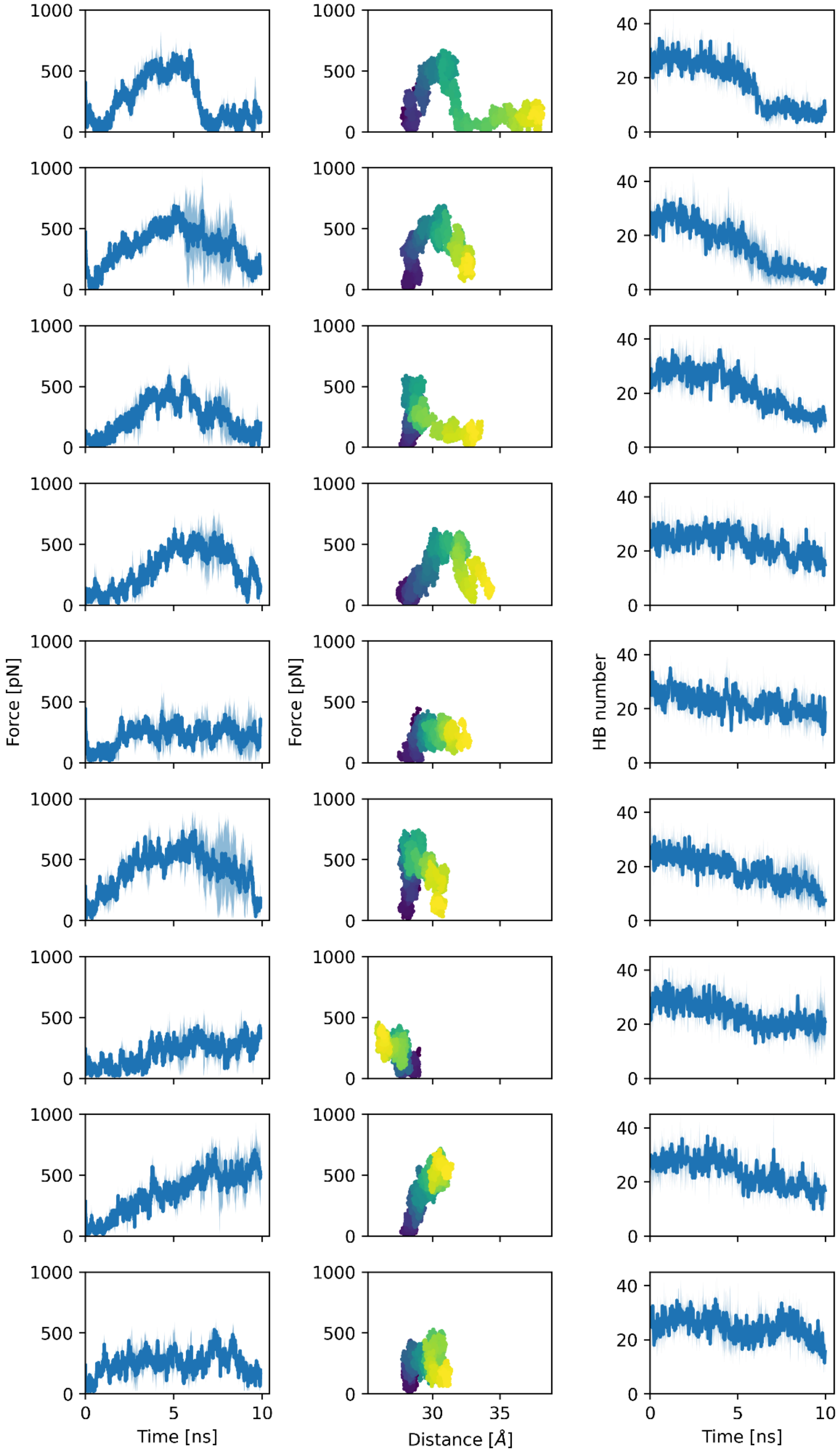

Figure S3: Example output from the multiSMD analysis script, showing the force variation over time (left), force versus distance between two selected anchor points (colored by simulation time progression, middle), and the time-dependent change in hydrogen bond count due to directional pulling (right). For the first and third columns, the

solid lines represent mean values, and the light-blue shaded areas (outline) represent the standard deviation (SD), both calculated from independent replica simulations for each pulling direction.

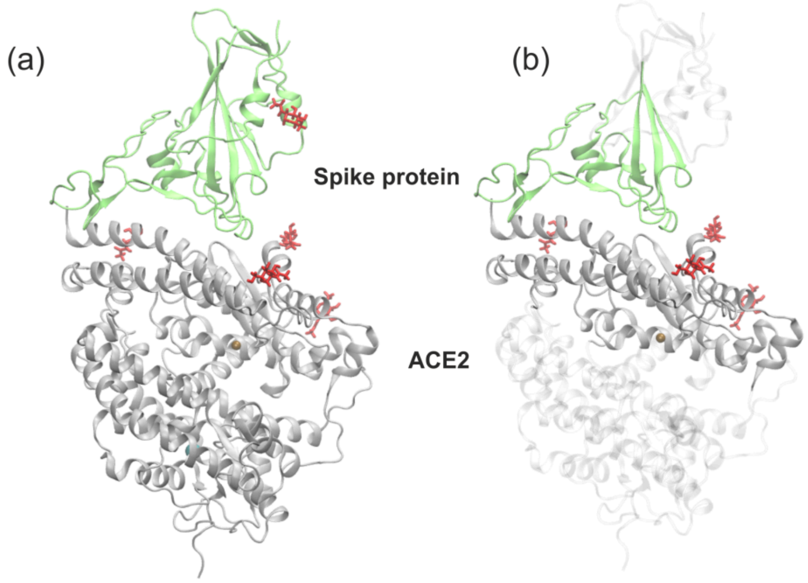

Figure S4: The truncated molecular system of the ACE2-S-protein complex used in our simulations. The ACE2 receptor is shown in gray and the viral spike protein is shown in green. Glycosylation sites are highlighted in red.

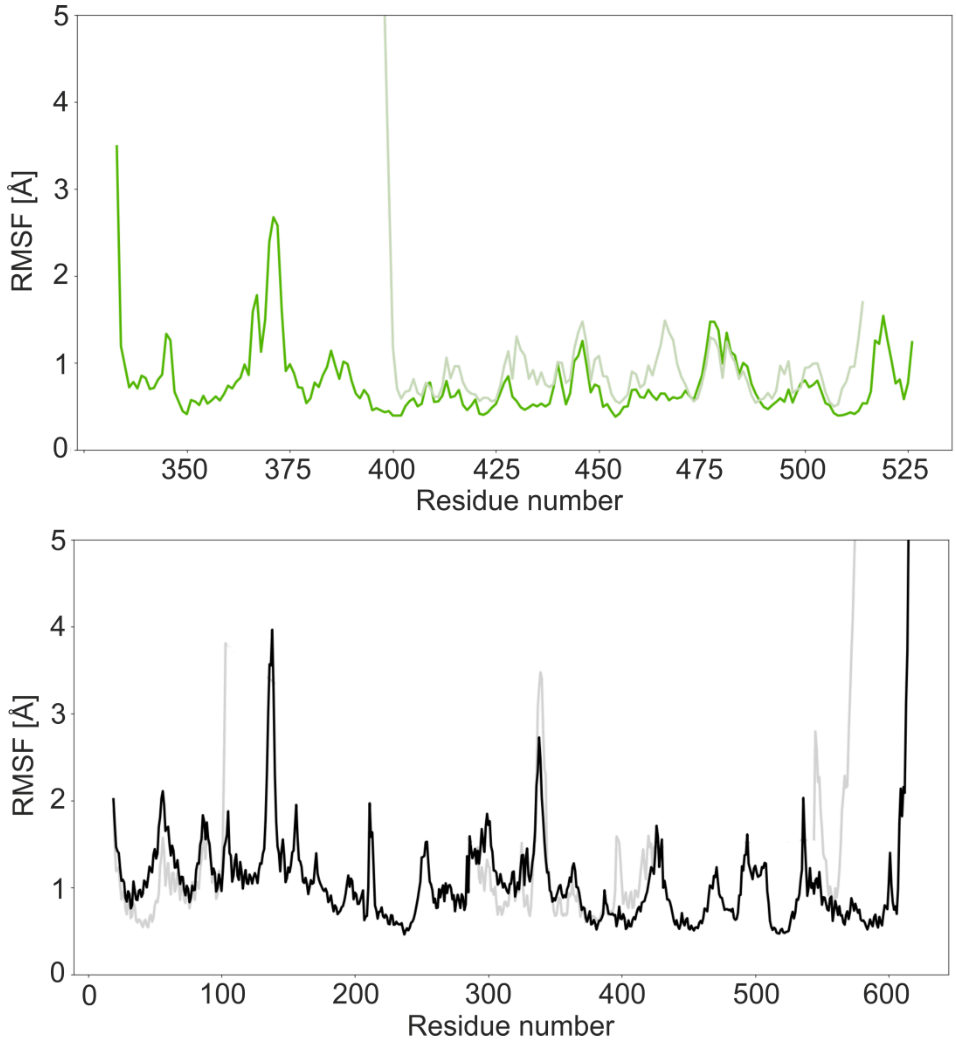

Figure S5: Root mean square fluctuations (RMSF) of the full system (gray) and truncated system (green and black) for the viral S-protein (upper panel) and ACE2 receptor (lower panel).

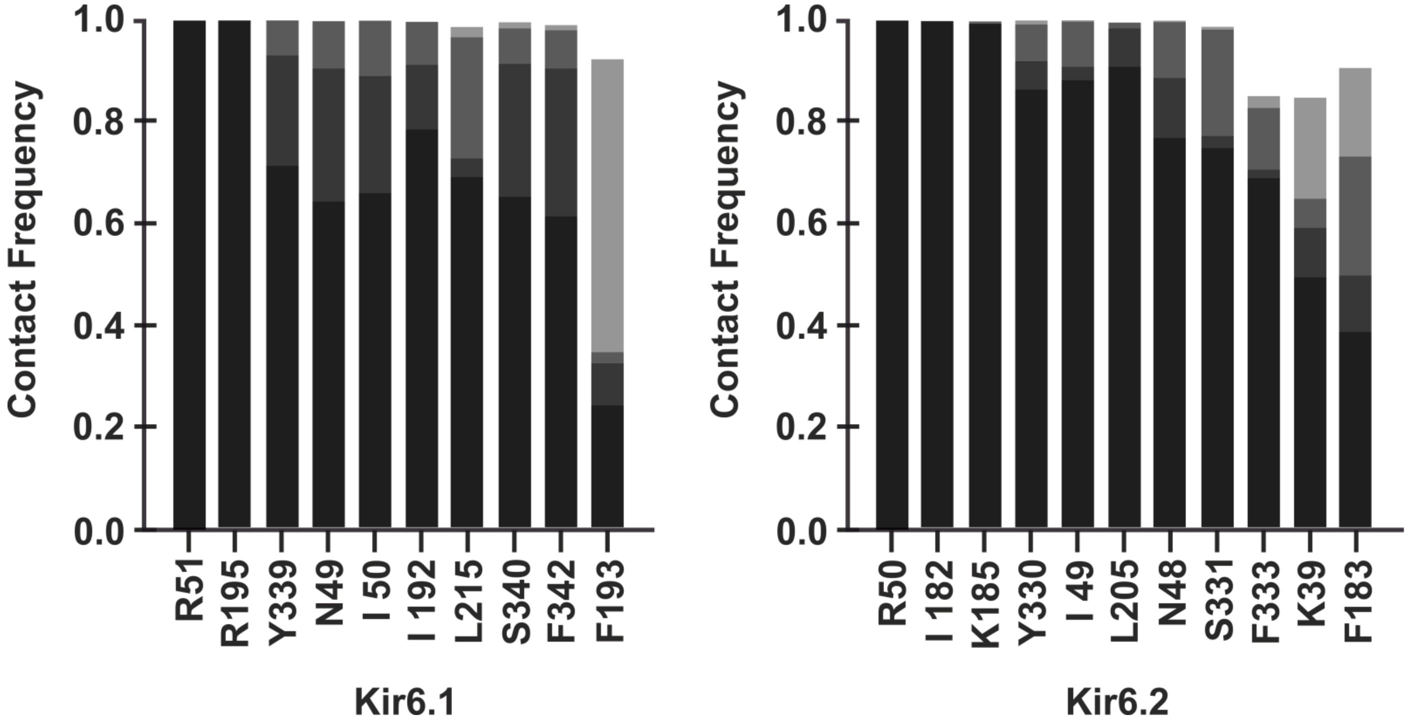

Figure S6: Frequency of close contacts between Kir6.1/Kir6.2 residues and ATP (docked at the inhibitory binding site) during unbiased molecular dynamics simulations.

References

1. Lan, J. *et al.* Structure of the SARS-CoV-2 spike receptor-binding domain bound to the ACE2 receptor. *Nature* **581**, 215–220 (2020).
2. Spinello, A., Saltalamacchia, A. & Magistrato, A. Is the Rigidity of SARS-CoV-2 Spike Receptor-Binding Motif the Hallmark for Its Enhanced Infectivity? Insights from All-Atom Simulations. *J Phys Chem Lett* **11**, 4785–4790 (2020).
3. Kern, N. R., Lee, J., Choi, Y. K. & Im, W. CHARMM-GUI Multicomponent Assembler for modeling and simulation of complex multicomponent systems. *Nat Commun* **15**, 5459 (2024).
4. Huang, J. *et al.* CHARMM36m: an improved force field for folded and intrinsically disordered proteins. *Nat Methods* **14**, 71–73 (2017).
5. Phillips, J. C. *et al.* Scalable molecular dynamics on CPU and GPU architectures with NAMD. *J Chem Phys* **153**, 044130 (2020).
6. Ye, F. *et al.* S19W, T27W, and N330Y mutations in ACE2 enhance SARS-CoV-2 S-RBD binding toward both wild-type and antibody-resistant viruses and its molecular basis. *Signal Transduct Target Ther* **6**, 343 (2021).
7. Driggers, C. M. & Shyng, S.-L. Mechanistic insights on KATP channel regulation from cryo-EM structures. *J Gen Physiol* **155**, (2023).
8. Patton, B. L., Zhu, P., ElSheikh, A., Driggers, C. M. & Shyng, S.-L. Dynamic duo: Kir6 and SUR in K channel structure and function. *Channels (Austin)* **18**, 2327708 (2024).

9. Sung, M. W. *et al.* Vascular K channel structural dynamics reveal regulatory mechanism by Mg-nucleotides. *Proc Natl Acad Sci U S A* **118**, (2021).

10. Quinn, K. V., Cui, Y., Giblin, J. P., Clapp, L. H. & Tinker, A. Do anionic phospholipids serve as cofactors or second messengers for the regulation of activity of cloned ATP-sensitive K+ channels? *Circ Res* **93**, 646–655 (2003).

11. Lee, K. P. K., Chen, J. & MacKinnon, R. Molecular structure of human KATP in complex with ATP and ADP. *Elife* **6**, (2017).

12. Vanoye, C. G. *et al.* The carboxyl termini of K(ATP) channels bind nucleotides. *J Biol Chem* **277**, 23260–23270 (2002).

13. Wu, J.-X. *et al.* Ligand binding and conformational changes of SUR1 subunit in pancreatic ATP-sensitive potassium channels. *Protein Cell* **9**, 553–567 (2018).

14. Martin, G. M. *et al.* Mechanism of pharmacochaperoning in a mammalian K channel revealed by cryo-EM. *Elife* **8**, (2019).

15. Walczewska-Szewc, K. & Nowak, W. Structural Determinants of Insulin Release: Disordered N-Terminal Tail of Kir6.2 Affects Potassium Channel Dynamics through Interactions with Sulfonylurea Binding Region in a SUR1 Partner. *J Phys Chem B* **124**, 6198–6211 (2020).

16. Sung, M. W. *et al.* Ligand-mediated Structural Dynamics of a Mammalian Pancreatic K Channel. *J Mol Biol* **434**, 167789 (2022).

17. Driggers, C. M., Kuo, Y.-Y., Zhu, P., ElSheikh, A. & Shyng, S.-L. Structure of an open K channel reveals tandem PIP binding sites mediating the Kir6.2 and SUR1 regulatory interface. *Nat Commun* **15**, 2502 (2024).
